# High-Performance Mixed Models Based Genome-Wide Association Analysis with omicABEL software

**DOI:** 10.12688/f1000research.4867.1

**Published:** 2014-08-20

**Authors:** Diego Fabregat-Traver, Sodbo Zh. Sharapov, Caroline Hayward, Igor Rudan, Harry Campbell, Yurii Aulchenko, Paolo Bientinesi

**Affiliations:** 1 Aachen Institute for Advanced Study in Computational Engineering Science, Aachen, 52062, Germany; 2 Institute of Cytology and Genetics, Siberian Division of the Russian Academy of Sciences, Novosibirsk, 630090, Russian Federation; 3 Novosibirsk State University, Novosibirsk, 630090, Russian Federation; 4 MRC Human Genetics Unit, Institute of Genetics and Molecular Medicine, University of Edinburgh, Edinburgh, EH4 2XU, UK; 5 Centre for Population Health Sciences, University of Edinburgh, Edinburgh, EH8 9AG, UK; 6 Split University, Split, 21000, Croatia

## Abstract

To raise the power of genome-wide association studies (GWAS) and avoid false-positive results in structured populations, one can rely on mixed model based tests. When large samples are used, and when multiple traits are to be studied in the ’omics’ context, this approach becomes computationally challenging. Here we consider the problem of mixed-model based GWAS for arbitrary number of traits, and demonstrate that for the analysis of single-trait and multiple-trait scenarios different computational algorithms are optimal. We implement these optimal algorithms in a high-performance computing framework that uses state-of-the-art linear algebra kernels, incorporates optimizations, and avoids redundant computations,

increasing throughput while reducing memory usage and energy consumption. We show that, compared to existing libraries, our algorithms and software achieve considerable speed-ups. The OmicABEL software described in this manuscript is available under the GNU

GPL v. 3 license as part of the GenABEL project for statistical genomics at http: //www.genabel.org/packages/OmicABEL.

## Introduction

Current biomedical research is experiencing a large boost in the amount of data generated. In particular, investigations involve human cohorts comprising hundreds of thousands of participants as part of nation-wide biobanking initiatives; furthermore, by using both arrays that include hundreds of thousands of single nucleotide polymorphisms (SNPs), and more recently, exome and whole-genome re-sequencing, the genomes of these participants are being characterized at an increasing level of detail, bringing the number of features assessed to tens of millions. At the same time, technologies for high-throughput assessment of different molecular “omics” phenotypes in large study cohorts are becoming more and more affordable. These molecular phenotypes characterize different classes and sub-classes of biological molecules, their functional modifications and relationships. Examples include hundreds of thousands of epigenetic modifications (epigenome ^1^), levels of tens of thousands of transcripts (transcriptome ^2, 3^), metabolites (metabonome or metabolome ^4, 5^), glycans (glyco(protein)ome ^6, 7^), and proteins (proteome ^8^). The evolution of current molecular techniques expands our capacity to access different components in the omics space, and new prominent omics emerge (e.g. cellomics, interactomics, activomics). The study of the genetic control of different omics brings the promise of new fundamental and applied biological discoveries; however, such analyses pose “big data” challenges.

Genome-Wide Association Studies (GWAS) is an established tool for analyzing the genetic control of complex traits ^9^. In GWAS, the association between millions of genetic markers (usually SNPs) and phenotype(s) of interest is studied, with significant associations highlighting the genomic regions harboring the functional variants involved in the control of the trait. While initially GWAS were mostly used to study common diseases, with the rising availability and affordability of omics phenotypes, this methodology is now also applied to investigate the omics space ^6, 7, 10– 13^, providing important insights into both the mechanisms underlying the genetic regulation of particular biological systems, and the determinants of human health and disease ^14– 16^.

In this work, we address the computational challenges posed by big-data GWAS. These challenges arise when size of sample under analysis is very large or when (potentially hundreds of) thousands of omics phenotypes are studied. We consider analyses facilitated by the use of linear mixed models (LMMs) ^17, 18^, which allow for modeling of correlations between phenotypes of relatives. The LMMs are among the most flexible and powerful methods to account for the genetic (sub)structure that inevitably occurs even in carefully designed large population-based studies. However, the increase in power and precision achieved through the use of mixed models comes with considerable costs in terms of computing time.

Recent advances in GWAS using mixed models ^19– 25^ represent a breakthrough compared to older methods, and allow analyses of a limited number of traits in reasonably sized samples even on personal computers. Still, current algorithms and software may be prohibitively expensive for analysis of large samples when dealing with omics data, since the time needed for a multi-trait analysis is essentially that of a one-trait study multiplied by the number of traits. Under this scenario, the analysis of even relatively small samples sizes leads to extremely long wait times. Therefore at the moment, the LMM-based GWAS analysis of large cohorts (tens to hundreds of thousands of participants), and even small (thousands of participants) studies involving omics measurements, represents a considerable problem. This limitation compromises the analyses availability, the data-to-knowledge turnaround time, and leads to excessive energy spending.

With this work, we aimed to address the aforementioned problems for big-data LMM-based GWAS. To do so, we took advantage of properties specific to the LMM formulation of GWAS, and analyzed a number of possible algorithms applicable to the analysis of large data. By combining sophisticated linear algebra and optimization techniques, we produced a fast and scalable software. Our software facilitates GWAS of tens of thousands of samples and hundreds of thousands of omics phenotypes, without the need for super-computing facilities.

## Methods

### Linear Mixed Models for GWAS

In a nutshell, LMM models the phenotypes of a group of *n* studied individuals as a point in an *n*-dimensional space, which comes from a multivariate Normal distribution. The expected mean is modeled using a standard regression model as *E*[ *Y*] = *Xβ*, where *X* is the design matrix which includes the genotypes of interest and other covariates, and *β* are fixed effects. The variance-covariance matrix is defined as *M* = *σ* ^2^ · ( *h* ^2^Φ + (1 *− h* ^2^) *I*); here, *σ* ^2^ is the total variance of the trait, *h* ^2^ is the heritability coefficient, *I* is the identity matrix, and Φ is the matrix containing the relationship coefficients for all pairs of studied individuals. GWAS are performed by consecutively including SNPs in the analysis model (usually one SNP at a time) and computing the association statistics for the included SNP, thus iteratively applying the model throughout the genome.

The statistical model considered in this work is the same as that outlined in previous works ^19– 21^, and proceeds with analysis in two steps: for each trait considered, we first estimate the matrix of (co)variances between phenotypes, and then we use it when estimating the SNP effects (see Supplementary Note S1 for mathematical details).

**Figure 1.**
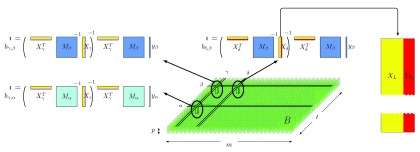
Interpretation of multi-trait GWAS as a two-dimensional grid of generalized least-squares problems ( *b* := ( *X ^T^ M* ^-1^ *X*) ^-1^ *X ^T^ M* ^-1^ *y*). GWAS with multiple phenotypes requires the solution of *m* × *t* correlated Generalized Least-Squares (GLS) problems, originating a three-dimensional object *B* of size *m* × *t* × *p*. Along the *t* direction, the variance-covariance matrix *M* and the phenotype *y* vary, while the design matrix *X* does not; conversely, in the *m* direction, *M* and *y* are fixed while *X* varies. Specifically, *X* can be viewed as consisting of two parts, *X _L_* and *X _R_*, where the former is constant across the entire grid and the latter changes along *m*. The figure also captures GWAS with single phenotype, in which case the dimension *t* reduces to 1.

Figure 1 illustrates how a multi-trait analysis consists of a series of *t* separate single-trait analyses, each of which, in turn, consists of a series of *m* Generalized Least-Squares (GLS) problems. The key to fast analysis algorithms is the realization that such problems are correlated, both along the *m* and the *t* direction; in big-data GWAS, any approach that ignores such correlations cannot be feasible in terms of time-to-solution.

### Efficient algorithms for single-trait and multi-trait GWAS

Aiming at supporting computational scientists in the design of efficient software, two of the authors recently developed CLAK, an algebraic system that replicates the reasoning of human experts for the automatic discovery of linear algebra solvers ^26^. The core idea is to first decompose a target matrix-based problem in terms of library-supported kernels, and then apply algorithmic and algebraic optimizations. Since the decomposition is not unique, CLAK returns not one but a family of possible solutions, all mathematically equivalent, but exhibiting different space and time complexity.

**Figure 2.**
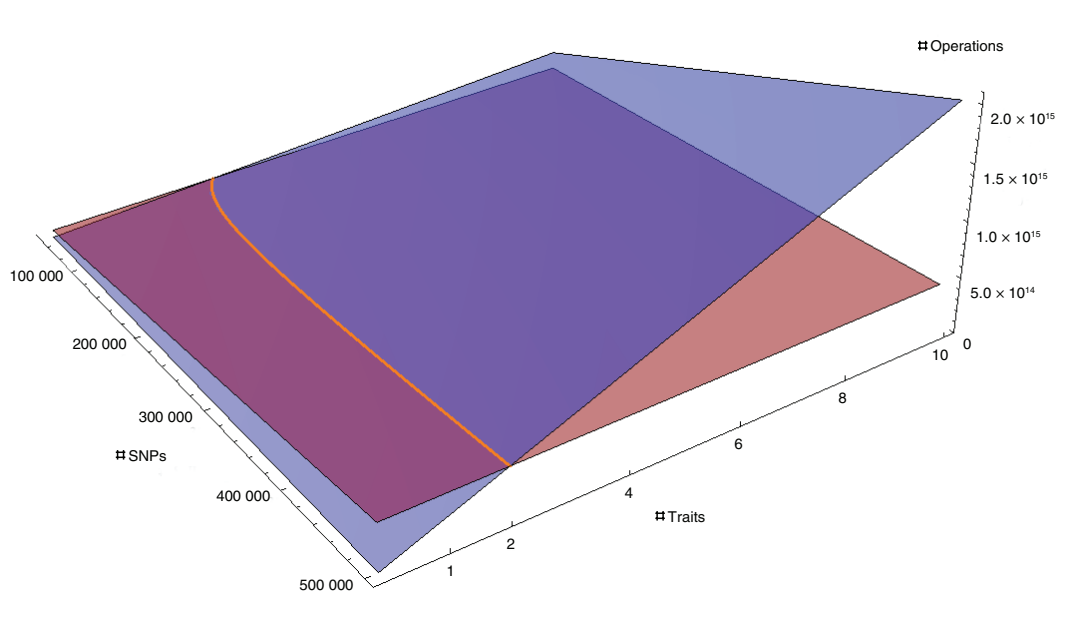
Cost analysis for CLAK-E ig and CLAK-C hol. The brown and blue surfaces indicate the number of operations performed by CLAK-E ig and CLAK-C hol, respectively, for a given number of SNPs and traits. The crossover curve suggests that for analyses with more than just a handful of traits, CLAK-E ig is the fastest algorithm.

With the help of CLAK, we generated many solutions to perform the aforementioned GWAS analyses (for a representative list, see Table S2). Each solution was subjected to the analysis of its computational complexity, expressing the cost in terms of the number of samples, markers, and traits in question. Interestingly, depending on the number of traits, the best theoretical performance was attained by two different solutions, which we named CLAK-C hol and CLAK-E ig (described in Table S1), respectively. Figure 2 shows the surfaces representing the time complexity of CLAK-C hol and CLAK-E ig as a function of the number of traits and markers analyzed; the solid curve denotes the crossover between the surfaces. When fewer than four traits are considered, CLAK-C hol attains better theoretical performance; on the contrary, for a higher number of traits, CLAK-E ig is expected to perform better (see also Table 1, and Supplementary Methods).

**Table 1.** Computational costs for the solution of single-trait and multiple-trait analyses. The variables *n*, *m* and *t* denote the sample size, the number of genetic markers, and the number of traits, respectively. *v* is the average number of iterations necessary to estimate the model parameters *σ* ^2^ and *h* ^2^ (see “Time complexity” in Supplementary Note S1).

Algorithm	Estimation of *σ* ^2^ and *h* ^2^	Single-trait analysis	Multi-trait analysis ( *t > n*)	Multi-trait analysis ( *n > t*)
CLAK-C hol	*O*( *n* ^3^ + *tvn*)	**O**( **mpn ^2^**)	*O*( *tmn* ^2^)	*O*( *tmn* ^2^)
CLAK-E ig	*O*( *n* ^3^ + *tvn*)	*O*( *mpn* ^2^)	**O**( **tmn**)	**O**( **mn ^2^**)
FaST-LMM	*O*( *n* ^3^ + *tvn*)	*O*( *mpn* ^2^)	*O*( *tmn* ^2^)	*O*( *tmn* ^2^)
GWFGLS	*O*( *n* ^3^ + *tvn*)	*O*( *mpn* ^2^)	*O*( *tmn* ^2^)	*O*( *tmn* ^2^)
EMMAX	*O*( *n* ^3^ + *tvn*)	*O*( *mpn* ^2^)	*O*( *tmn* ^2^)	*O*( *tmn* ^2^)

The idea underlying CLAK-C hol is to linearly transform the input data to de-correlate the observations. To this end, the variance-covariance matrix *M* is formed explicitly, and its triangular Cholesky factor *L* is computed ( *LL ^T^* = *M*); through this factor, each SNP *X* and each trait *y* is then linearly transformed, giving raise to a sequence of Ordinary Least Squares problems of the form *b* := ( *X ^T^X*) ^-1^ *X ^T^y*. While such problems are solvable with standard techniques, CLAK-C hol takes advantage of the fact that the covariates are fixed for all SNPs, hence lowering the computational complexity. In single-trait analyses, it is also possible to exploit the fact that the matrix *M* is symmetric and positive definite. Although asymptotically the Cholesky factorization is equivalent to an eigen-decomposition, in practice it requires 10 times fewer operations. Moreover, instead of using the eigenvectors (a full matrix) to rotate the SNPs, the Cholesky factor (a triangular matrix) allows us to transform them at half the cost. For more details, see the work we have previously published ^27^ describing the CLAK-C hol algorithm.

The design of CLAK-E ig is based on three insights: 1) For a given study, the relationship matrix Φ is constant across both SNPs and traits; 2) since the variance-covariance matrix *M* is built by merely shifting and scaling relationship matrix, its eigenvectors are the same as those of Φ, and its eigenvalues are obtained by shifting and scaling those of Φ; 3) the inverse of *M* is easily expressed by inverting the diagonal matrix containing its eigenvalues (note that in our solutions we never explicitly invert matrices; we instead factor them, and operate with their factors). Together, these insights suggest that the eigen-decomposition of Φ can be computed once and for all, and most importantly, the eigenvectors of the relationship matrix can be used to rotate all the SNPs and traits only once. After the data is rotated, the computation of the mixed model based GWAS can be carried out by means of a grid of inexpensive Weighted Least Squares problems.

Once the initial eigen-decomposition of Φ is available, the complexity of CLAK-E ig is determined by three operations: the rotation of the SNPs, the rotation of the traits, and the solution of the Weighted Least Squares problems. The dominant term depends on the size of population ( *n*), number of SNPs ( *m*), and number of traits ( *t*). When *n > t* (or *n > m*), the overall time complexity comes from the rotation of the SNPs (or the traits), and amounts to *O*( *n* ^2^ *m*) (or *O*( *n* ^2^ *t*)); if instead both *t* and *m* are larger than *n*, then the dominant term comes from the Least Squares problems, and is linear in population, SNPs and traits: *O*( *nmt*) ( Table 1). Note that the CLAK-E ig algorithm is a generalization of the eigen-decomposition based algorithms published before (e.g. ^22, 28^) for a case of multiple trait analysis.

Compared with current state-of-the-art algorithms ^19, 21, 22^ in multi-trait analyses, CLAK-E ig achieves a lower computational complexity. As shown in Table 1, there are two scenarios of interest, depending on whether the number of traits is larger than the population size or not. In the first case ( *t > n*), which is probably the most typical for current and near-future omics studies, the time complexity of CLAK-E ig is linear on the number of markers, traits, and samples; by contrast, all the other methods have quadratic complexity with the sample size. In the second case ( *n > t*), which takes place for smaller ‘omics’ and also will become more common with the increasing affordability of omics technologies and hence larger sample sizes, the cost of CLAK-E ig is determined by the sample size and the number of SNPs, and its complexity is a factor *t* lower than other methods.

For both CLAK-E ig and CLAK-C hol, the space complexity is mainly determined by the square of the sample size; also, a minimum of one trait, one SNP, and the *p* covariates must reside in memory. In total, our methods only require enough memory to accommodate *n* ^2^+(2+ *p*) *n* entries. If multiple SNPs and/or traits fit in main memory at once, —e.g., dozens or hundreds of them—the computational throughput of our methods improves noticeably. In this case, the space requirement becomes *n* ^2^+( *k*+ *p*) *n*, where *k* is the number of SNPs and traits resident in memory. As examples, for sample sizes of 10,000, 20,000 and 40,000, the *n* ^2^ space requirement translates to 1, 3, and 12 GBs, respectively. More details on space complexity are provided in Supplementary Note S1.

## Results

### Implementation and comparison

To demonstrate the practical advantages of CLAK-E ig and CLAK-C hol, we implemented these algorithms in the O micABEL software package. In doing so, we tailored our implementations to save intermediate results across adjacent problems; we also re-organized the calculations to fully benefit from both the efficiency of highly optimized linear algebra kernels, and the parallelism offered by modern computing platforms.

Since the size of the datasets involved in GWAS is considerably larger than the memory capacity of current processors, input and output data can only be stored in disk devices. Aware that the penalty for accessing information residing on disk is enormous—several orders of magnitude greater than the cost for performing one arithmetic operation—it is imperative to handle these big-data efficiently. By means of asynchronous transfers between memory and disk, our algorithms achieve a perfect overlap of computation and data movement. As long as the relationship matrix fits in the main memory, and regardless of the size of the data sets—both in terms of SNPs and phenotypes—, the processor never idles waiting for a transfer to complete, thus computing at maximum efficiency.

**Figure 3.**
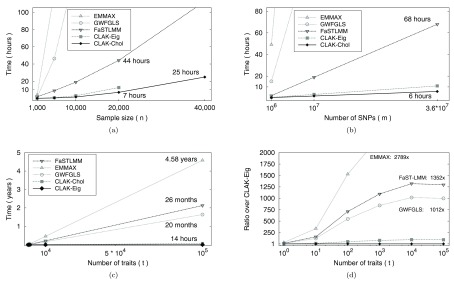
Timings comparison. Panels ( **a**) and ( **b**) include timings for EMMAX, GWFGLS, FaST-LMM, and our O micABEL software, for single trait analyses; ( **c**) and ( **d**) present a comparison of EMMAX, GWFGLS, FaST-LMM, and our O micABEL software, in the case of multiple traits. In ( **a**), the number of SNPs is fixed to *m* = 10 ^7^ and the sample size *n* ranges from 1,000 to 40,000. In panel ( **b**), the sample size is fixed to *n* = 10,000 and the number of SNPs *m* ranges between 10 ^6^ and 3.6 × 10 ^7^. In ( **c**) and ( **d**), *n* = 1,000, *m* = 10 ^6^ and *t* ranges from 1 to 100,000.

We compared the GWAS run-time of CLAK-C hol and CLAK-E ig as implemented in O micABEL with that of several well-established packages: EMMAX ^19^, FaST-LMM ^22^ (two-step approximation), and GWFGLS (implementation of the mmscore method of ProbABEL ^21^ in the MixABEL-package ^29^). In the experiments, we considered three different scenarios, varying one among sample size *n*, number of SNPs *m*, and number of traits *t*, while keeping the other two values constant ( Figure 3). A description of the experimental setup is provided in Supplementary Note S2 and Supplementary Note S3.

In the first scenario (single trait and *m* = 10,000,000 SNPs; the sample size varies from 1,000 to 40,000), all methods exhibit a quadratic behavior, and CLAK-C hol is the only algorithm that completed all tests within 25 hours ( Figure 3(a)). For the largest problem considered (sample size *n* = 40,000), the speed-up over the next-fastest software (FaST-LMM) is 8.3: 205 vs. 25 hours; when *n* = 1,000, the speed-ups over GWFGLS, FaST-LMM and EMMAX are 24, 32 and 68, respectively: 86, 112 and 240 vs. 3.5 minutes.

The second scenario (single trait and sample size of 10,000; the number of markers varies between 1 and 36 millions) shows a linear dependence on the number of genetic markers for all software packages. Again, CLAK-C hol attains the best timings, outperforming FaST-LMM, GWFGLS and EMMAX by a factor of 11.7, 93.6 and 298, respectively ( Figure 3(b)), when the number of analysis SNPs is 36 millions.

Finally, the third scenario illustrates the analysis of multiple phenotypes (sample size of 1,000 and 1,000,000 genetic markers; number of traits varies between 1 and 100,000). The (estimated) time required for these analyses is presented in Figure 3(d). Note that the time scale on this graph is in years. Due to CLAK-E ig’s linear time complexity with respect to sample size, SNPs and traits, its advantage becomes most apparent: when thousands of traits are considered, CLAK-E ig outperforms GWFGLS, FaST-LMM and EMMAX by a factor of 1012, 1352, and 2789, respectively ( Figure 3(d)), bringing the execution time down from months to hours.

### Demonstration of application to real data

We applied the O micABEL (CLAK-E ig) to study 107,144 metabolomic traits in a sample of 781 people from a genetically isolated population of the Vis island (Croatia). These data are part of the EUROSPAN data set reported in the works of 11, and 12. In short, the data comprise plasma levels of 23 sphingomyelins (SPM), 9 ceramides (CER), 56 phosphatidylcholines (PC), 15 lysophosphatidylcholines (LPC), 27 phosphatidylethanolamines (PE), and 19 PE-based plasmalogens (PLPE). From these data, additional traits were defined by aggregating species into groups with similar characteristics (e.g. unsaturated ceramides), and also by expressing data as molar percentages (instead of absolute concentrations) within classes. Following the standards accepted in genetic analysis of metabolomics data ^30^, in this work, 328 such measurements served as a base to compute all pair-wise ratios, resulting in 107,584 traits, which were analyzed for association with 266,878 SNPs. More details about the data are provided in Supplementary Note S4.

Previously, it took several weeks to accomplish the original analysis of only few hundreds “original” traits. However, using our O micABEL software and a computer with 40 cores, we were able to finish the analysis of more than 100,000 traits in only 8 hours.

Table S4 shows the results for five SNPs previously reported ^11^ to be significantly associated with levels of circulating sphingolipid concentrations. The best results obtained for these SNPs when using the original and derived traits are reported. The implicated traits were also analyzed by using other approaches: the full likelihood ratio test based LMM ^18, 22^ (as implemented in MixABEL::FMM), Grammar- *γ* ^25^ (as implemented in the GenABEL-package ^29^), and the two-step approach ^19– 21^ (as implemented in MixABEL::GWFGLS). From Table S4 one can see that our results are consistent with those obtained by other methods. Additionally, Table S3 summarizes the results concerning heritabilities of analyzed traits, while Table S5 lists the Geinflation factor *λ* obtained when analyzing the selected traits with different methods. More details of the analysis of this human metabolomics data set are provided in Supplementary Note S4.

## Discussion

In contemporary human genomics, methods and tools face the additional challenge posed by the sheer size of the datasets. Big data are produced from the investigation of large human cohorts including hundreds of thousands of participants; these massive samples facilitate the identification of small effects and lead to important biological insights. Large data also come from the field of functional (gen)omics, which aims at establishing the functional roles of genetic variants; hence GWAS are increasingly applied not only to study complex traits in large cohorts, but also to understand the regulation of human and animal transcriptome ^15, 31, 32^, metabolome ^10– 12^, glyco(protein)ome ^6, 7^ and other types of omics data. Results of these studies are used to uncover the link between these molecular phenotypes and high-level complex traits, including human diseases ^14– 16^.

 In recent years, linear mixed model was accepted as a powerful tool for whole-genome analysis of genetic associations ^17, 18^. Most current LMM-based methods for GWAS ^19– 24^ exhibit linear dependency of the compute time on the number of genetic polymorphisms and traits studied, but at least quadratic dependency on the sample size. A notable exception from the latter “at-least-quadratic” rule is the GRAMMAR-Gamma method ^25^ and a method based on low rank approximation of the similarity matrix ^22^ - with the latter exploiting the ideas similar to EIGENSTRAT approach ^33^ and the methods assuming the adjustment of the model for top Principal Components of the kinship matrix variation. However, the computational advantage of these methods comes at the cost of mathematical approximation. For example, the GRAMMAR-Gamma method, while extremely fast, and showing excellent results for human studies, is less suited for analyses of samples with uneven genetic structure; adjusting for top principle components (and EIGENSTRAT) is known to provide incomplete correction for stratification in case of complex kinship. Increasing sample sizes and availability of molecular omics phenotypes lead to “big data challenges” and the computational throughput of LMM’s starts being more and more of an issue, which at present sometimes cannot be resolved without resorting to supercomputing facilities.

With this work, we address the problem of mixed-model based whole-genome analysis of genetic association for an arbitrary number of traits. We describe the CLAK-C hol and CLAK-E ig algorithms and software tools (O micABEL package) to address LMM-based GWAS. Specifically, our CLAK-C hol will be useful for investigation of complex traits in very large (tens of thousands of individuals) samples, while CLAK-E ig will be a useful tool for the investigation of genetic control of different omics, potentially including hundreds of thousands or even millions of features.

As for our CLAK-C hol approach, we are not aware of similar, Cholesky-based solutions proposed before. The CLAK-E ig approach behind our solutions bear similarities, and actually reduces to previously suggested methods (e.g. ^22, 28^) when the number of traits is one. It is also worth mentioning the Matrix eQTL software ^34^, which, while not implementing the LMM, in many respects exploits the problem-specific properties of multi-trait GWAS in the ways similar to ours.

The key achievement of this research is that it facilitates big-data LMM-based GWAS without supercomputers. For a sample problem with a population of 1,000, three covariates, one million SNPs and 100,000 traits, we estimate that the available methods would require the entire Sequoia supercomputer (equipped with 1.5 million cores) ^1^ for about 3 minutes; by contrast, using a common 40-core compute node (see Supplementary Methods, Note S2), our method completes within a day and reduces the energy consumption by a factor of 200 (estimated). It should be noted that this impressive speed-up comes at the price of additional assumption of complete data. For many types of omics assays assumption of absence of missing data could be (almost) true, and a small proportion of missing data could be imputed (in the simplest case – replaced with average value) with little negative effect onto statistical properties of the method. However, for the omics assays which produce large proportion of missing data, our CLAK-E ig method in its current formulation and implementation would be inapplicable, unless the missing values could be reliably imputed.

In case of single-trait analysis, our results are somewhat less impressive, and our CLAK-C hol solution outperforms advanced current methods (e.g. FaST-LMM) by about one order of magnitude for large-sample-size problems. It is worth mentioning that the latter speed-up becomes possible because we show that for single-trait GWAS problems our CLAK-C hol algorithm is superior to CLAK-E ig, but other current methods are actually implementing solutions similar to our CLAK-E ig algorithm to address the single-trait GWAS problem.

Further optimizations of our solutions are possible, for instance by exploiting the structure of the kinship matrix. A “compressed MLM” approach was proposed for decreasing the effective sample size of datasets by clustering individuals into groups ^20^; similarly, the fast decaying and possibly sparse structure of the kinship matrix can be exploited to lower the number of mathematical operations. Caution must be exercised in the interpretation of findings resulting from GWAS analyses as they may generate false positives if the multiple testing problem is not addressed adequately. A conservative strategy to determine whether an association is statistically significant would be to apply a Bonferronni correction, that is, in our example analysis of 107,144 traits, the conventional genome-wide significance threshold *p*-value of 5 · 10 ^-8^ should be replaced by 4.7 · 10 ^-13^. This is the common approach applied in the metabolomics studies (see ^10, 30^). On the other hand, this threshold would probably be too conservative, given that many of the measurements may be highly correlated. Several methods have been introduced recently ^35– 37^, which may help to overcome this problem; however, this topic lays outside the scope of the current work.

For the analysis of specific omics data, our methods (and software) might require some modifications. For example, in the genetic regulation of human transcriptome, large attention is dedicated to cis-eQTLs, computationally a relatively simple task. In contrast, our implementation is tailored to perform full GWAS for every trait analyzed. While O micABEL could be used for the identification of trans-eQTLs, one should be aware of the specifics of the analysis of this type of data (e.g. allele-specific expression in RNAseq studies) and a body of methods developed (e.g. methods to account for influences of hidden factors ^38, 39^).

We foresee that the primary use of our algorithms and software is within the domain of analysis of complex traits in very large samples and for the genetic analysis of “omics” data. However, potentially, there are other uses. The same set of methods and tools can be used for scanning through other omics in e.g. search for biomarkers for a complex trait or in order to determine functional relations between different omics. For example, one may be interested in doing epigenome-wide scans relating the epigenome to a complex trait (or other omics, such as metabolome). Under this use, the genomic inputs would be replaced by epigenomic data. Advanced statistical and machine learning methods, such as penalized regression, can make use of joint analysis of up to several hundreds of thousands of predictors ^40^. One of the common scenarios include the construction of millions of features, and their filtering for further joint analysis—a task which can be also effectively addressed by our methods. Finally, our algorithms can be easily extended, e.g. to search for interactions.

## Conclusions

We demonstrated that different computational algorithms are optimal for the problems of single- and multi-trait Mixed-Model based GWAS, and implemented these algorithms in a freely available O micABEL software.

## Software availability

### Software access

The O micABEL software implementing computational methods described in this manuscript is available as part of the G enABEL project for statistical genomics at http://www.genabel.org/packages/OmicABEL. The web-page provides the link to O micABEL tutorial, giving examples of its use.

### Latest source code

https://r-forge.r-project.org/scm/viewvc.php/pkg/OmicABEL/?root=genabel

### Archived source code as at the time of publication

http://dx.doi.org/10.5281/zenodo.10999 ^41^

### Software license

GNU GPL v. 3

## Figures and Tables

**Table S1.** The CLAK-C hol and CLAK-E ig algorithms for single-trait and multi-trait analyses, respectively.

CLAK-C hol: Single-trait analysis		CLAK-E ig: Multi-trait analysis
1 $\overset{̭}{M}$ := $\overset{̭}{\sigma }$ ^2^( $\overset{̭}{h}$ ^2^Φ + (1 – $\overset{̭}{h}$ ^2^) *I*) 2 *LL ^T^* = $\overset{̭}{M}$ 3 *X _L_* := *L* ^–1^ *X _L_* 4 *X _R_* := *L* ^–1^ *X _R_* 5 *y* := *L* ^–1^ *y* 6 *S _TL_* := *X ^T^_L_ X _L_* 7 *b _T_* := *X ^T^_L_ y* 8 **for** i = 1:m 9 $S\;\text{:=}\;\left(\frac{S_{TL}\;|\;\;*}{X_{R_{i}}^{T}X_{L}|X_{R_{i}}^{T}X_{R_{i}}}\right)$ 10 $b_{i\;}\;\text{:=}\;\left(\frac{b_{T}}{X_{R_{i}}^{T}y}\right)$ 11 *b _i_* := *S*_*i*_^–1^ *b _i_*		1 *ZWZ ^T^* = Φ 2 *X _L_* := *Z ^T^ X _L_* 3 *X _R_* := *Z ^T^ X _R_* 4 *Y* := *Z ^T^ Y* 5 **for** j = 1: t 6 *D* := ( $\overset{̭}{\sigma }$ ^2^_j_( $\overset{̭}{h}$ ^2^_j_ *W* + (1 – $\overset{̭}{h}$ ^2^_j_) *I*)) ^–1^ 7 *KK ^T^* = *D* 8 *Y _j_* := *K ^T^ Y _j_* 9 *W _L_* := *K ^T^ X _L_* 10 *S _TL_* := *W ^T^_L_ W _L_* 11 *b _T_* := *W ^T^_L_ Y _j_* 12 **for** i = 1:m 13 *W _R_* := *K ^T^ X _R _i__* 14 $S\;\;\text{:=}\;\left(\frac{S_{TL}\;|\;\;\;\;\;\;*}{W_{R}^{T}W_{L}|W_{R}^{T}W_{R}}\right)$ 15 $b_{ij}\;\text{:=}\;\left(\frac{b_{T}}{W_{R}^{T}Y_{j}}\right)$ 16 *b _ij_* := *S* ^–1^ *b _ij_*

**Table S2.** Representative list of algorithms for solving a single generalized least-squares problem, as generated by the CLAK expert system.

CLAK-C hol’s for a single GLS problem		CLAK-E ig’s for a single GLS problem
1 $\overset{̭}{M}$ := $\overset{̭}{\sigma }$ ^2^( $\overset{̭}{h}$ ^2^Φ + (1 – $\overset{̭}{h}$ ^2^) *I*) 2 *LL ^T^* = $\overset{̭}{M}$ 3 *X* := *L* ^–1^ *X* 4 *y* := *L* ^–1^ *y* 5 *S* := *X ^T^X* 6 *b* := *X ^T^y* 7 *b* := *S* ^–1^ *b*		1 *ZWZ ^T^* = Φ 2 *D* := ( $\overset{̭}{\sigma }$ ^2^( $\overset{̭}{h}$ ^2^ *W* + (1 – $\overset{̭}{h}$ ^2^) *I*)) ^–1^ 3 *KK ^T^* = *D* 4 *X'* := *Z ^T^X* 5 *y'* := *Z ^T^y* 6 *V* := *K ^T^X'* 7 *ν* := *K ^T^y'* 8 *S* := *V ^T^V* 9 *b* := *V ^T^ν* 10 *b* := *S* ^–1^ *b*
Algorithm 7		Algorithm 13
1 $\overset{̭}{M}$ := $\overset{̭}{\sigma }$ ^2^( $\overset{̭}{h}$ ^2^Φ + (1 – $\overset{̭}{h}$ ^2^) *I*) 2 *LL ^T^* = $\overset{̭}{M}$ 3 *X* := *L* ^–1^ *X* 4 *QR* := *X* 5 *y* := *L* ^–1^ *y* 6 *b* := *Q ^T^y* 7 *b* := *R* ^–1^ *b*		1 *ZWZ ^T^* = Φ 2 *D* := ( $\overset{̭}{\sigma }$ ^2^( $\overset{̭}{h}$ ^2^ *W* + (1 – $\overset{̭}{h}$ ^2^) *I*)) ^–1^ 3 *KK ^T^* = *D* 4 *X'* := *Z ^T^X* 5 *V* := *K ^T^X'* 6 *QR* := *V* 7 *y'* := *L* ^–1^ *y* 8 *ν* := *K ^T^y'* 9 *b* := *Q ^T^ν* 10 *b* := *R* ^–1^ *b*

**Table S3.** Regression analysis of heritability estimates in different traits classes. The label “Original” refers to the average heritability of the original traits (intercept of the model); similarly, “cer-cer” refers to the heritability of ratios involving CER, “cer-lpc” to the heritability of ratios involving CER and LPC, and so on.

Trait	Estimate	Std. Error	t value	Pr(> | *t*|)
Original	0.310493	0.007645	40.613	< 2e-16
cer-cer	0.126283	0.009268	13.625	< 2e-16
cer-lpc	0.009870	0.008183	1.206	0.22777
cer-pc	0.012576	0.007833	1.606	0.10838
cer-pe	-0.016897	0.007982	-2.117	0.03426
cer-pls	-0.008811	0.008176	-1.078	0.28119
cer-spm	0.067942	0.008095	8.393	< 2e-16
lpc-lpc	0.009306	0.008364	1.113	0.26590
lpc-pc	-0.035843	0.007769	-4.614	3.96e-06
lpc-pe	-0.053195	0.007868	-6.761	1.38e-11
lpc-pls	-0.024212	0.008001	-3.026	0.00248
lpc-spm	-0.021573	0.007942	-2.716	0.00660
pc-pc	-0.062170	0.007732	-8.041	9.03e-16
pc-pe	-0.063491	0.007722	-8.222	< 2e-16
pc-pls	-0.059816	0.007767	-7.702	1.36e-14
pc-spm	-0.044365	0.007749	-5.725	1.04e-08
pe-pe	-0.041556	0.007922	-5.246	1.56e-07
pe-pls	-0.056304	0.007864	-7.160	8.13e-13
pe-spm	-0.049549	0.007834	-6.325	2.54e-10
pls-pls	-0.056809	0.008341	-6.811	9.75e-12
pls-spm	-0.039083	0.007941	-4.922	8.60e-07
spm-spm	-0.010163	0.008164	-1.245	0.21319

**Table S4.** Nominal *p*-values for the analysis of SNPs previously associated with circulating sphingolipid concentrations in Vis data. *Lead trait*: the original trait for which the association signal was most significant in Vis data. *Lead percentage*: the molar percentage, for which the most significant association was obtained. *Lead Ratio*: the ratio, for which the most significant association was obtained. OmicA: OmicABEL, our implementation of CLAK-E ig; FASTA: the two-step approach ^19– 21^ (as implemented in MixABEL::GWFGLS); LRT: the full likelihood ratio test based LMM ^18, 22^ (as implemented in MixABEL::FMM); Gra- *γ*: Grammar- *γ* ^25^ (as implemented in the GenABEL-package ^29^).

SNP	Trait	Method			
		OmicA	FASTA	LRT	Gra- *γ*
	Original traits				
rs10938494 rs1000778 rs4902242 rs7258249 rs680379	PC 40:6 PC 38:4 SPM 14:0 SPM 18:1 C23:0	3.83E-03 4.07E-06 3.42E-13 3.93E-08 9.90E-05	3.66E-03 3.49E-06 2.70E-13 3.71E-08 9.53E-05	3.71E-03 3.14E-06 1.03E-13 2.42E-08 8.69E-05	3.89E-03 3.39E-06 2.75E-13 3.97E-08 9.77E-05
	Molar percentages				
rs10938494 rs1000778 rs4902242 rs7258249 rs680379	% PC 40:6 % PC 36:4 % SPM 14:0 % SPM 18:1 % C16:0	8.42E-03 2.90E-04 1.32E-13 2.30E-05 9.43E-04	8.25E-03 2.92E-04 1.11E-13 2.33E-05 8.91E-04	8.04E-03 2.29E-04 4.08E-14 1.92E-05 8.61E-04	8.25E-03 2.30E-04 1.19E-13 2.35E-05 9.07E-04
	Ratios				
rs10938494 rs1000778 rs4902242 rs7258249 rs680379	SPM 24:1 / glucosylceramide % SPM 23:0 / % PC 36:4 SPM 14:0 / % SPM 23:0 SPM 16:1 / SPM 18:1 SPM 16:1-OH / % C16:0	2.29E-06 8.83E-09 1.71E-15 6.41E-08 1.56E-07	1.72E-06 8.16E-09 1.35E-15 5.72E-08 1.52E-07	1.31E-06 6.04E-09 4.44E-16 3.74E-08 1.17E-07	1.89E-06 7.64E-09 8.95E-15 6.23E-08 1.60E-07

**Table S5.** Genomic Control *λ* estimates for traits analyzed in Table S4 (main text).

Trait	Method			
	OmicA	FASTA	LRT	Gra- *γ*
Original traits				
PC 40:6 PC 38:4 SPM 14:0 SPM 18:1 C23:0	0.995 0.987 0.992 0.998 0.995	1.008 1.000 0.999 1.005 1.004	0.999 1.001 0.996 1.014 0.999	0.989 1.026 0.998 0.995 0.993
Molar percentages				
% PC 40:6 % PC 36:4 % SPM 14:0 % SPM 18:1 % C16:0	1.007 0.964 0.995 0.998 0.989	1.015 0.995 1.005 1.002 1.001	1.006 1.006 1.004 1.002 1.008	0.985 1.010 0.993 0.994 0.991
Ratios				
SPM 24:1 / glucosylceramide % SPM 23:0 / % PC 36:4 SPM 14:0 / % SPM 23:0 SPM 16:1 / SPM 18:1 SPM 16:1-OH / % C16:0	0.975 1.008 1.006 1.008 0.997	1.005 1.007 1.009 1.010 1.002	0.998 1.006 0.997 0.999 1.003	0.994 1.011 0.994 0.994 0.993

## Methods and algorithms

### Mixed Models for GWAS

The Variance Components model for a quantitative trait can be formulated as

Y = Xβ+R,

where *Y* is the vector containing the phenotypes for *n* individuals, *X* is the design matrix, and *β* and *R* are the vectors of fixed and random effects, respectively. The partitioning *X* = [1| *L*| *g*] indicates that the design matrix is composed of three parts: 1 denotes a column-vector (corresponding to the intercept) containing ones, *L* is an *n × p* matrix corresponding to fixed covariates such as age and sex, and *g* typically consists of a single column-vector containing genotypes. The vector of random effect *R* is assumed to be distributed as a Multivariate Normal with mean zero and variance-covariance matrix *M* = *σ* ^2^( *h* ^2^Φ + (1 − *h* ^2^) *I*); here, *σ* ^2^ is the total variance of the trait, *h* ^2^ (in the range [0, 1]) is the heritability coefficient, *I* is the identity matrix, and Φ is the matrix containing the relationship coefficients for all pairs of studied individuals. The relationship matrix Φ can be estimated from the pedigree or from the genomic data ^18^.

In GWAS, the quantity of interest is the effect of the genotype, that is, the element(s) of *β* corresponding to *g*. Technically, a GWAS with mixed model consists of traversing all measured polymorphic sites in the genome, substituting the corresponding *g* into *X*, and fitting the above model; the result is millions of estimates of genetic effect together with their *p*-values.

One of the most used mixed model-based approaches used in GWAS relies on a two-step analysis methodology ^19– 22, 41^. In the first step, the reduced model (with *X* = [1| *L*]) is fit to the data, thus obtaining the estimates { $\overset{̭}{\sigma }$ ^2^, $\overset{̭}{h}$ ^2^}; the variance-covariance matrix corresponding to such estimates is denoted by $\overset{̭}{M}$ = $\overset{̭}{\sigma }$ ^2^( $\overset{̭}{h}$ ^2^Φ + (1 – $\overset{̭}{h}$ ^2^) *I*). In the second step, for each *g _i_* and corresponding *X _i_* = [1| *L*| *g _i_*], the estimates of the effects and the variance-covariance matrix are respectively obtained as

$${\overset{̭}{\beta }}_{i}={\left(X_{i}^{T}{\overset{̭}{M}}^{-1}X_{i}\right)}^{-1}X_{i}^{T}{\overset{̭}{M}}^{-1}y,\;\;\;\;\;(1)$$

and

$$Var\left({\overset{̭}{\beta }}_{i}\right)={\overset{̭}{\sigma }}^{2}{\left(X_{i}^{T}{\overset{̭}{M}}^{-1}X_{i}\right)}^{-1},$$

with *i* = 1, . . . , *m*, where *m* is the number of genetic markers considered.

In this work, we consider an extended formulation of this problem to the case of multiple phenotypes, that is, Y is a collection of *t* vectors, with *y _j_* ( *j* = 1, . . . , *t*) being a vector corresponding to a specific trait. In this case, trait-specific estimates $\overset{̭}{\sigma }$ ^2^_j_, $\overset{̭}{h}$ ^2^_j_ need to be obtained, resulting in *t* different $\overset{̭}{M}$ *_j_*s. As the result of the analysis, *m × t* vectors of estimates of $\overset{̭}{\beta }$ *_ij_* and corresponding *Var*( $\overset{̭}{\beta }$ *_ij_*) are generated. In summary, the problem we are facing is

$$\left\{ \begin{array}{l} \;\;\;{\overset{̭}{\beta }}_{ij}={\left(X_{i}^{T}{\overset{̭}{M}}_{j}^{-1}X_{i}\right)}^{-1}X_{i}^{T}{\overset{̭}{M}}_{j}^{-1}y_{j}\\ Var({\overset{̭}{\beta }}_{ij})={\overset{̭}{\sigma }}_{j}^{2}{\left(X_{i}^{T}{\overset{̭}{M}}_{j}^{-1}X_{i}\right)}^{-1}\;\;\;\;\;\;\;\;\;\text{with}\;1\leq i\leq m\;\;\;\;\;(2)\\ \;\;\;{\overset{̭}{M}}_{j}={\overset{̭}{\sigma }}_{j}^{2}\left({\overset{̭}{h}}_{j}^{2}\Phi +\left(1-{\overset{̭}{h}}_{j}^{2}\right)I\right)\;\;\;\;\;\;\text{and}\;1\leq j\leq t; \end{array}\right.$$

see Figure 1 for a visual description. For each *i* and *j*, Equation (2) represents a generalized least-square (GLS) problem. Single-trait and multiple-trait analyses correspond to the solution of *m×*1 (1-dimensional) and *m×t* (two-dimensional) grids of GLS problems, respectively.

### Single-instance algorithms

We provide here an overview of a simplified version of CLAK-C hol and CLAK-E ig to solve a single GLS; the versions tailored for one-dimensional and two-dimensional grids of GLS’s are discussed in the Methods section and presented in Table S1. In the following, we use *b* to indicate $\overset{̭}{\beta }$; in both our algorithms, the computation of *Var*( *b*) represents an intermediate result towards *b*.

***CLAK-Chol***. The approach for CLAK-C hol ( Table S2, top-left) is to first reduce the initial GLS *b* := ( *X ^T^* $\overset{̭}{M}$ ^–1^ *X*) ^–1^ *X ^T^* $\overset{̭}{M}$ ^–1^ *y* to a linear least-squares problem, and then solve this via normal equations. Specifically, the algorithm starts by forming $\overset{̭}{M}$ = $\overset{̭}{\sigma }$ ^2^( $\overset{̭}{h}$ ^2^Φ + (1 – $\overset{̭}{h}$ ^2^) *I*), which is known to be symmetric positive definite, and by computing its Cholesky factor *L*. This leads to the expression *b* := ( *X ^T^ L ^−T^ L* ^−1^ *X*) ^–1^ *X ^T^* *L* ^− *T*^ *L* ^−1^ *y*, in which two triangular linear systems can be identified and solved— $\overset{ˍ}{X}$ := *L* ^−1^ *X* and $\overset{ˍ}{y}$ := *L* ^−1^ *y* —thus completing the reduction to the standard least-squares problem *b* := ( $\overset{ˍ}{X}$ *^T^* $\overset{ˍ}{X}$) ^–1^ $\overset{ˍ}{X}$ *^T^* $\overset{ˍ}{y}$. Numerical considerations allow us to safely rely on the Cholesky factorization of *S* := $\overset{ˍ}{X}$ *^T^* $\overset{ˍ}{X}$ without incurring instabilities. The algorithm completes by computing $\overset{ˍ}{b}$ := $\overset{ˍ}{X}$ *^T^* $\overset{ˍ}{y}$ and solving the linear system *b* := *S* ^–1^ $\overset{ˍ}{b}$, for a total cost of $\frac{1}{3}$ *n* ^3^ + *O*( *n* ^2^ *p*).

***CLAK-Eig***. Instead of forming the matrix $\overset{̭}{M}$, Algorithm CLAK-E ig ( Table S2, top-right) operates on the matrix Φ: At first, it diagonalizes Φ as *ZWZ ^T^*, leading to the expression

$$\overset{̭}{M}\;\text{:=}\;{\overset{̭}{\sigma }}^{2}({\overset{̭}{h}}^{2}ZWZ^{T}+(1-{\overset{̭}{h}}^{2})I),$$

with diagonal *W*. By orthogonality of *Z*, the inverse of $\overset{̭}{M}$ can be represented as

$${\overset{̭}{M}}^{-1}\;\text{:=}\;Z{\left({\overset{̭}{\sigma }}^{2}({\overset{̭}{h}}^{2}W+(1-{\overset{̭}{h}}^{2})I)\right)}^{-1}Z^{T}$$

and easily computed with a cost of *O*( *n*) operations via

$$D\;\text{:=}\;{\left({\overset{̭}{\sigma }}^{2}({\overset{̭}{h}}^{2}W+(1-{\overset{̭}{h}}^{2})I)\right)}^{-1};$$

the solution to the GLS is thus given by

$$b\;\text{:=}\;{\left(X^{T}ZD^{-1}Z^{T}X\right)}^{-1}X^{T}ZD^{-1}Z^{T}y.\;\;\;\;(3)$$

Moreover, since *D* is symmetric positive definite, Equation 3 can be rewritten as

$$b\;\text{:=}\;{\left(X^{T}ZKK^{T}Z^{T}X\right)}^{-1}X^{T}ZKK^{T}Z^{T}Y,$$

and the algorithm proceeds by computing *V* := *X ^T^ZK* (matrix-matrix multiplication and scaling), obtaining *b* := ( *VV ^T^*) ^–1^ *VK ^T^Z ^T^y*. Similar to CLAK-C hol, *b* is finally obtained through matrix-vector multiplications and a linear system, for a total cost of $\frac{10}{3}$ *n* ^3^ + *O*( *n* ^2^ *p*).

### Effectively solving the grids of Generalized Least-Squares problems

As shown in Table 1, the strength of the algorithms CLAK-C hol and CLAK-E ig becomes apparent in the context of 1D and 2D grids of GLS problems, corresponding to GWAS with single and multiple phenotypes, respectively. The straightforward approach, which is the only alternative provided by current general-purpose numerical libraries, lies in a loop that utilizes the best performing algorithm for a single least-square problem. No matter how optimized the GLS solver, such an approach is prohibitively expensive for either single or multiple phenotypes, due to the unmanageable complexity of *O*( *mn* ^3^) and *O*( *tmn* ^3^), respectively. By contrast, the versions of CLAK-C hol and CLAK-E ig shown in Table S1 are the product of a number of optimizations aimed at saving intermediate results across successive problems, thus avoiding redundant calculations. For instance, the matrix *X* is logically split as [ *X _L_|X _R_*], where *X _L_* includes the intercept and the covariates ([1| *L*]), while *X _R_* is the collection of all the genetic markers *g _i_*. Thanks to these savings, both algorithms achieve a lower overall complexity.

Unfortunately, the reduced complexity of algorithms CLAK-C hol and CLAK-E ig is not enough to guarantee high-performance implementations. It is well known that in terms of execution time, the difference between a straightforward translation of an algorithm into code and a carefully assembled routine is of at least one order of magnitude. In other words, the benefits inherent to our new algorithms might go unnoticed unless paired with state-of-the-art implementation techniques. In this following, we detail our strategy to attain high-performance routines.

Both CLAK-C hol and CLAK-E ig are entirely expressed in terms of standard linear algebra operations, such as matrix products and matrix factorizations, provided by the BLAS ^42^ and LAPACK ^43^ libraries. Since LAPACK itself is built in terms of BLAS kernels, these are the main responsible for the overall performance of an algorithm. BLAS consists of a relatively small set of highly optimized kernels, organized in three levels (1, 2, and 3), corresponding to vector, matrix-vector, and matrix-matrix operations, respectively.

A common misconception is that all the BLAS kernels, across the three levels, attain a comparable (and high) level of efficiency. Instead, it is only BLAS-3—when operating on large matrices—that fully exploits the processors’ potential; as an example, the matrix-vector multiplication, matrix-matrix multiplication on small matrices, and matrix-matrix multiplication on large matrices attain an efficiency of *≈* 5%, *≈* 15%, and more than 95%, respectively. In this context, the linear systems *X* := *L* ^−1^ *X* in CLAK-C hol (Line 3 of the top-left algorithm in Table S2), to be solved for each individual SNP, should ideally be aggregated into a single—very large—linear system *X _R_* := *L* ^−1^ *X _R_*, in which *X _R_* is the collection of the genetic markers of all SNPs (Line 3 of the left Algorithm in Table S1). An analogous comment is valid for CLAK-E ig (see the multiplications at Lines 3 and 4 of the right Algorithm in Table S1), in which the number of markers accessed at once is a user-configurable input parameter.

Since all the current computing platforms include multicore processors, we briefly discuss how to take advantage of this architecture. An effective practice is to invoke a multi-threaded version of the BLAS library. Both in CLAK-C hol and CLAK-E ig ( Table S1) we rely on such a solution for the sections leading up to the outer loop (Lines 1–7 and 1–4, respectively). In the remainder of the algorithms (Lines 8–11 and 5–16), due to the lack of matrix-matrix operations, we instead apply a thread-based parallelization in conjunction with single-threaded BLAS. This mixed use of multi-threading becomes more and more effective as the number of available computing cores increases.

### Time complexity

Prior to solving a grid of GLS, the heritability ( *h* ^2^) and the total variance ( *σ* ^2^) have to be estimated. For this first step, our algorithms use an approach similar to Software packages such as GenABEL ^29^, EMMAX, and FaST-LMM: First, the eigen-decomposition of the kinship matrix is performed, for a computational cost of *O*( *n* ^3^). Then, the model parameters are estimated using an iterative procedure based on the Maximum Likelihood (GenABEL, FaST-LMM) or Restricted Maximum Likelihood (EMMAX, CLAK-C hol, CLAK-E ig) methods, for a cost of *O*( *tvnp* ^2^), where *v* is the average number of iterations required to reach convergence. In total, the parameter estimation has a complexity of *O*( *n* ^3^ + *tvnp* ^2^) operations.

In the second step, a 1D or 2D grid of GLS is solved, corresponding to single and multiple phenotype analysis. For 1D grids, all the considered methods share the same asymptotic time complexity, but the constant factor for CLAK-C hol is the lowest, yielding a speedup factor of at least 6. In 2D grids, EMMAX, FaST-LMM and GenABEL simply tackle each individual trait independently, one after another, for a total complexity of *O*( *tmpn* ^2^). By exploiting the common structure of the variance-covariance matrix of different phenotypes, CLAK-E ig reduces the complexity by a factor of *n*, down to *O*( *tmpn*). As a result, our algorithm outperforms the other methods by factors higher than 1,000.

### Space requirement

***CLAK-C hol***. To form and factor the variance matrix, the algorithm uses *n* ^2^ memory (Lines 1–2 in the left Algorithm of Table S1). The overall space requirement is determined by the triangular solve (Line 4), which necessitates the full *L* and a portion of *X* to reside in memory at the same time. This operation is performed in a streaming fashion—operating on *k* SNPs at the time—and overwriting *X* . All the other instructions do not require any extra space. In total, CLAK-C hol uses about *n* ^2^ + *kn* memory.

***CLAK-E ig***. The initial eigen-decomposition (Line 1 in the right Algorithm of Table S1) needs 2 *n* ^2^ memory. The following matrix-matrix multiplications (Lines 2, 3 and 8) overwrite the input matrices and again are performed in a streaming fashion; in terms of space, the analysis is similar to that for the triangular solve in CLAK-C hol. The remaining instructions do not affect the overall memory usage. In total, the space requirement is 2 *n* ^2^ + *kn*.

If memory is a limiting factor, one can set *k* to 1 in either algorithm, possibly at the cost of exposed data movement. Moreover, by using a different (slower) eigensolver, the eigenvectors *Z* can overwrite the input matrix Φ, effectively saving *n* ^2^ memory. These considerations indicate that our algorithms are capable of solving GWAS of any size, as long as the kinship matrix, one SNP, and one trait fit in RAM.

## Computing environment

All the computing tests were run on a symmetric multiprocessor system consisting of four Intel Xeon E7-4850 10-core processors, operating at a frequency of 2.00 GHz. The system is equipped with 256GB of RAM and 4TB of disk as secondary memory ^2^. The routines were compiled with the GNU C Compiler (gcc v4.4.5) and linked to Intel’s MKL multi-threaded library (v12.1). For double-buffering, our out-of-core routines make use of the AIO library, one of the standard libraries on UNIX systems. The available multi-core parallelism is exploited through MKL’s multi-threaded BLAS and OpenMP’s pragma directives.

## Simulated data

A data set for GWAS can be characterized by the number of individuals in the sample ( *n*), the number of measured and/or imputed SNPs ( *m*) to be tested, the number of outcomes to be analyzed ( *t*), and the number of covariates ( *p*) to be included in the model. In current GWAS, a typical scenario consists of a few covariates (for example, two, such as sex and age), 10 ^5^–10 ^7^ SNPs, and thousands or tens of thousands individuals. In our experiments, we assumed that the number of covariates is two (p=2), and we varied the three other characteristics ( *n*, *m*, *t*) of the data set, leading to these three scenarios.

(A) The number of SNPs was fixed to *m* = 10,000,000 and one single outcome ( *t* = 1) was studied. As sample size, we used 1,000, 5,000, 10,000, 20,000, and 40,000. The latter test represents a scenario with large number of individuals.

(B) The number of individuals was fixed to *n* = 10,000 and one single outcome ( *t* = 1) was studied. The number of markers *m* to be analyzed was set to 1,000,000, 10,000,000, and 36,000,000. The latter test is a scenario that represents a whole genome re-sequencing.

(C) The number of individuals and markers were fixed to *n* = 1,000 and *m* = 1,000,000, respectively. The number of outcomes *t* studied varied (1, 10, 100, 1,000, 10,000, and 100,000), corresponding to an Omics analysis.

For testing purposes, we generated artificial data sets which met pre-specified values of *t*, *m*, *p*, and *n*.

## Application to human metabolomics data

### CROATIA-Vis study

The CROATIA-Vis study includes 1056 unselected Croatians, aged 18–93 years, who were recruited into the study during 2003 and 2004 from the villages of Vis and Komiza on the Dalmatian island of Vis ^44, 45^. The settlements on Vis island have unique population histories and have preserved their isolation from other villages and from the outside world for centuries. Participants were phenotyped for more than 450 disease-related quantitative traits. Biochemical and physiological measurements were performed, detailed genealogies reconstructed, questionnaire of lifestyle and environmental exposures collected, and blood samples and lymphocytes extracted and stored for further analyses. Samples in all studies were taken in the fasting state. The CROATIA-Vis study was approved by the ethics committee of the medical faculty in Zagreb and the Multi-Centre Research Ethics Committee for Scotland.

### Metabolomics measurements

We applied the OmicABEL to study 107,144 metabolomic traits in a sample of 781 people from a genetically isolated population of the Vis island (Croatia). These data are a part of the EUROSPAN data set reported in work of Hicks ^11^ and Demirkan and colleagues ^12^. In short, the data comprise plasma levels of 23 sphingomyelins (SPM), 9 ceramides (CER), 56 phosphatidylcholines (PC), 15 lysophosphatidylcholines (LPC), 27 phosphatidylethanolamines (PE), and 19 PE-based plasmalogens (PLPE). From these data, additional traits were then defined by aggregating species into groups with similar characteristics (e.g. unsaturated ceramides), and also by expressing data as molar percentages (instead of absolute concentrations) within classes. In this work, 328 such measurements served as a base to compute all pair-wise ratios, resulting in 107,584 traits. The traits with *>*95% of measured values without ties were sex- and age-adjusted and then Gaussianized using the quantile normalization. The resulting 107,144 traits were subject to GWAS with 266,878 SNPs.

Here we report several findings showing the power of this approach to large-scale hypothesis-free analysis; while such an analysis was hardly conceivable before, is now within the reach for most researchers by using our new methods, algorithms and software.

### Results

In total, heritability was *>*0 for 102,985 (96.1%) traits, with an average heritability of 27%, and a maximum of 85%. We investigated if there were systematic differences in heritabilities between different classes of measurements. The average heritability of the original traits was 0.31 (see Table S3). We observed that heritabilities were specific for different classes of ratios. For example, the ratios involving ceramides were more heritable than the “original” traits, especially for the ratios CER:CER ( *h* ^2^_*CER:CER*_ = 0.44 vs. *h* ^2^_*orginal*_ = 0.31, *p <* 10 ^–15^) and CER:SPM ( *h* ^2^_*CER:SPM*_ = 0.38, *p <* 10 ^–15^). All ratios involving LPC, PC, PE, PLS, and SPM had significantly lower (all *p <* 0.01) heritabilities, except for the cases when CER’s were involved in the ratio or, in the biologically plausible case in which the ratios were derived within the same lipids class (i.e. LPC:LPC, PE:PE, etc.).

To this end, we re-analyzed the associations for five SNPs previously reported ^11^ to be significantly associated with levels of circulating sphingolipid concentrations. Table S4 reports the best results obtained for these SNPs when using the original and derived traits. The traits implicated were also analyzed by using the full likelihood ratio test based LMM (MixABEL::FMM), Grammar- *γ* ^25^, and MixABEL::GWFGLS (which is very similar to OmicABEL) methods. All *p*-value were corrected for the Genomic Control inflation factor (presented in Table S5). From Table S4 one can see that our results are consistent with these obtained by other methods.

Note that we have analyzed only a subset of the data reported by Hicks *et al.* ^11^, and therefore our lead concentrations did not always come from the class reported in previous work (e.g. *ATP10D* was reported to be associated with glucosylceramides and *FADS* with SPM).

## Notes

^1^As of January of 2014, Sequoia is the third fastest supercomputer in the world: .

^2^Recall that our algorithms do not require large amounts of available RAM, as long as it accommodates the kinship matrix, the algorithms will complete.
