# Supplementary material for: High-Performance Mixed Models Based Genome-Wide Association Analysis with omicABEL software
Source: F1000Res. 2014 Aug 20;3:200. [Version 1] doi: 10.12688/f1000research.4867.1 (PMC4329600; doi:10.12688/f1000research.4867.1)
Supplement: Peer review history

# Peer review: Referee response for version 1

**DOI:** 10.5256/f1000research.5195.r5915

**Published:** 2015-02-11

**Authors:** Bertram Muller-Myhsok

**Affiliations:** 1 Max Planck Institute for Psychiatry, Munich, Germany

This is a very well written report on a very important topic of very high impact in the actual of omics and multi-level omics data, allowing beyond sheer analysis (which in large data sets is very demanding indeed in terms of runtime and memory) many more questions answered also for methods research.

The methods put forward present a very major step forward for this type of analysis. The methodology is very sound and promising. A tool to actually use is provided, which is also not always the case.

The authors are to be commended for this work.

I have read this submission. I believe that I have an appropriate level of expertise to confirm that it is of an acceptable scientific standard.

# Peer review: Referee response for version 1

**DOI:** 10.5256/f1000research.5195.r5916

**Published:** 2014-09-05

**Authors:** Peter Holmans

**Affiliations:** 1 MRC Center for Neuropsychiatric Genetics and Genomics, Cardiff University, Cardiff, UK

This article introduces a new software tool (omicABEL) for performing rapid association analyses between SNP data and high-dimensional "omics" data (e.g. gene expression) using mixed models. This is an extremely important development for the following reasons:

1. Identification of genetic variants associated with gene expression (eQTLs), methylation (mQTLs) etc is becoming ever more important in understanding the biology of complex genetic disorders.
2. It is desirable, when performing association analyses, to allow for population stratification and/or cryptic relatedness among members of the sample. Mixed models are an effective way of doing this.
3. The high dimensionality of the "omics" data (tens or even hundreds of thousands of measurements) has made the application of mixed model association approaches computationally prohibitive up to now. In addition to detailing an application of considerable practical utility, the article is also clearly written and mathematically rigorous.

I have read this submission. I believe that I have an appropriate level of expertise to confirm that it is of an acceptable scientific standard.

# Peer review: Referee response for version 1

**DOI:** 10.5256/f1000research.5195.r5981

**Published:** 2014-08-29

**Authors:** Dirk Jan de Koning

**Affiliations:** 1 Department of Animal Breeding and Genetics, Swedish University of Agricultural Sciences, Uppsala, Sweden

The manuscript shows a computational approach that enABELs the use of linear mixed mixed models for large pedigrees, large numbers of markers and large numbers of (omics) traits.

While the work is interesting, several things need to be clarified before the work can be of interest to the wider community.

I assume that the manuscript is aimed at the potential users: researchers in genetics with an interest to analyze their 'big data' in a correct fashion and in a repeatable fashion. The manuscript is compact but possibly too compact in places. I would like the following points to be addressed:

 

1. Accuracy of estimates and comparison of results. The authors make a big deal about comparing the approaches in terms of computing time. They should also be compared systematically in terms of type I error, accuracy of estimates etc. The computational approximations that are made must come at some cost and as researchers we need to know this cost. There are some token comparisons in the supplementary tables but these deal with heritability and P values. Not with the actual SNP effects which are what we want from these analyses. Some scatter plots between different methods may be helpful.
2. We need more detail about the simulations. You simulate different population sizes but you must clarify the family structures and the family complexity. How many generations? All family sizes equal? etc. Also how you simulate marker data in terms of historical population size, lD structure etc. Likewise for correlation structure among the multiple traits.
3. In many places, variables are used before they are defined. p appears in Table 1 but is only clarified later in the text. The tables are currently not readable on their own and the acronyms and variables should be explained within the tables.

 

In general, the mathematical notation as well as the language in general needs a bit of work. Some sentences are too long with too many clauses, for example CLAK is not defined at first use. X and y are linearly transformed: should you not introduce new variables for the transformed data?

- Some parts of result should be in the Materials and Methods.
- Figure 3C could have a logarithmic Y-axis.
- Figure 3D could omit line for CLAK-Eig and CLAK-Chol is unlabeled.
- The abbreviation in Table S3 cer-cer lpc, pls spm pc pe etc. makes no sense to me. They are different trait classes but still the table adds very little.

 

The manuscript has the promise to introduce an interesting new computational approach, but we need more details to make up our mind.

I have read this submission. I believe that I have an appropriate level of expertise to confirm that it is of an acceptable scientific standard, however I have significant reservations, as outlined above.
